# Right Ventricular Failure and Pathobiology in Patients with Congenital Heart Disease – Implications for Long-Term Follow-Up

**DOI:** 10.3389/fped.2013.00037

**Published:** 2013-11-19

**Authors:** Doreen Köhler, Raoul Arnold, Tsvetomir Loukanov, Matthias Gorenflo

**Affiliations:** ^1^Department of Pediatric Cardiology, University of Heidelberg, Heidelberg, Germany; ^2^Department of Cardiac Surgery, Division of Congenital Cardiac Surgery, University of Heidelberg, Heidelberg, Germany

**Keywords:** right ventricle, congenital heart defects, imaging, surgical techniques, pathobiology

## Abstract

Right ventricular dysfunction represents a common problem in patients with congenital heart defects, such as Tetralogy of Fallot or pulmonary arterial hypertension. Patients with congenital heart defects may present with a pressure or volume overloaded right ventricle (RV) in a bi-ventricular heart or in a single ventricular circulation in which the RV serves as systemic ventricle. Both subsets of patients are at risk of developing right ventricular failure. Obtaining functional and morphological imaging data of the right heart is technically more difficult than imaging of the left ventricle. In contrast to findings on mechanisms of left ventricular dysfunction, very little is known about the pathophysiologic alterations of the right heart. The two main causes of right ventricular dysfunction are pressure and/or volume overload of the RV. Until now, there are no appropriate models available analyzing the effects of pressure and/or volume overload on the RV. This review intends to summarize clinical aspects mainly focusing on the current research in this field. In future, there will be increasing attention to individual care of patients with right heart diseases. Hence, further investigations are essential for understanding the right ventricular pathobiology.

## Right Ventricular Failure in Congenital Heart Disease

In patients with congenital heart disease (CHD), the right ventricle (RV) serves in a bi-ventricular heart as subpulmonal ventricle or in a single ventricular circulation as the systemic ventricle. RV dysfunction is a common problem in the clinical care for patients with CHD. Clinical relevance for research projects in the field of RV dysfunction is given by the following examples:

*Tetralogy of Fallot after intracardiac repair*: Volume overload of the RV due to pulmonary regurgitation results in RV dilatation and RV dysfunction. Significant RV dilatation with an end-diastolic volume >170 ml/m^2^ or an endsystolic volume >85 ml/m^2^ is associated with persistent RV dilatation even after successful surgical pulmonary valve replacement by using a valved homograft (1). Other patients with Tetralogy of Fallot develop a “restrictive RV physiology,” which is associated with decreased cardiac output after cardiac surgery and the need for prolonged intensive care.

*Univentricular heart with RV predominance*: Morphology and function of the systemic ventricle (right versus left) are important factors influencing the long-term prognosis after Fontan type (TCPC, total cavopulmonary connection) repair. Precisely, RV morphology of the systemic ventricle is associated with a much worse outcome and RV dysfunction is associated with higher mortality and morbidity rates in this group of patients (2).

*Right ventricular outflow tract obstruction* is observed in numerous congenital heart defects (pulmonary valve stenosis, double-chambered RV, etc.). Long persisting RV outflow tract obstruction (RVOTO) will finally lead to systolic and diastolic RV dysfunction.

After atrial repair using the Senning or Mustard techniques in patients with *d-transposition of the great arteries* (d-TGA) the RV serves as the systemic ventricle. The survival rate of these patients has been reported with 76% at 20 years of age, the mean age at death was 27 years (3). The function of the systemic RV determines the outcome of these patients (4). The same holds true for unoperated patients with AV- and VA-discordance (so called congenitally corrected transposition of the great arteries, *ccTGA*). In both conditions, adolescents and young adults are prone to RV failure which finally necessitates orthotopic heart transplantation (5).

While the mechanisms of left ventricular dysfunction have been well examined, there is only very limited information available concerning various aspects of RV dysfunction in the setting of chronic volume and/or pressure overload. In contrast, given the low overall mortality of congenital cardiac surgery in neonates and infants, more and more patients will survive into adulthood when there is an increased incidence of RV dysfunction. Therefore, the function of the RV is the major determinant of morbidity and mortality. Nonetheless, precise data on the incidence of RV failure in patients with CHD are lacking. Patients presenting with the RV as the systemic ventricle and patients after surgical repair for tetralogy of Fallot and pulmonary valve insufficiency have the highest risk for developing heart failure (6). In 2006, the “National Heart, Lung, and Blood Institute” (National Institutes of Health, Bethesda, MD, USA) therefore addressed the top priority for research on the RV pathophysiology.

Several disease entities predispose to the development of heart failure as a consequence of a dysfunctional RV. In patients after repair of Fallot tetralogy, severe pulmonary regurgitation will lead to RV dilatation and – if not treated adequately – also to left ventricular dysfunction (6). The risk to develop heart failure is even higher in patients with single ventricles after Fontan procedure (7), especially in patients with the RV serving as systemic ventricle, who have the highest risk for RV dysfunction (8).

Cardiac resynchronization therapy (CRT) has been shown to improve mortality and morbidity in adult patients with refractory heart failure and prolonged QRS duration. In children and CHD patients treated with CRT heart failure symptoms have been found to improve at a mean follow-up of 0.7 years (9). However long-term data are lacking and it remains a major challenge to define potential candidates in the subset of patients with CHD and heart failure who will benefit from CRT as pediatric data from controlled trials are lacking (10).

Currently, stem cell therapy with cardiac progenitor cells (CPCs) is under evaluation in clinical trials (11). Rupp and coworkers could impressively show first results of stem cell therapy in children. In 2010, an infant with hypoplastic left heart syndrome was reported to improve after successful intracoronary administration of autologous bone marrow-derived progenitor cells (12–14).

Taking these features and the consequences for lifelong care for these patients into account, there is a need to initiate research projects addressing the pathophysiology of the RV under the conditions of pressure and/or volume overload.

## Challenges of Right Ventricular Imaging

Imaging of the RV aims at obtaining morphological and functional data. The anatomy of the RV makes it more difficult to achieve imaging data on muscle mass as well as ventricular volumes during systole and diastole compared to the left ventricle. Magnetic resonance imaging (MRI) has gained wide acceptance to evaluate RV morphology and function. Imaging of the RV nowadays aims at giving prognostic information for patients with RV volume and/or pressure overload. In patients with a systemic RV, the combination of a RV end-diastolic volume index above 150 ml/m^2^ (by MRI or multi-detector computed tomography imaging) and data from exercise tests (peak exercise systolic blood pressure below 180 mmHg) was found to identify patients with a 20-fold higher annual event rate compared to patients without these risk factors (15).

The right and left ventricle strongly interact. Therefore it is impossible to view abnormalities of the RV without taking the left ventricle into account (16), what had also been shown by an animal model of RV dilatation caused by ischemia. The occlusion of the right coronary artery with intact pericardium resulted in a decrease in LV end-diastolic volume and contractility which was the result of acutely altered RV geometry (17).

**Figure 1:**
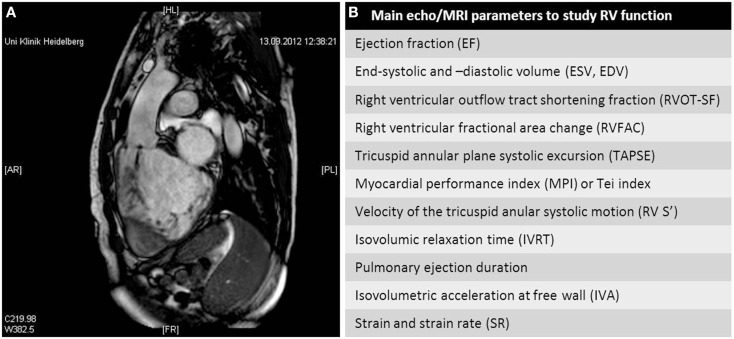
**(A)** Magnetic resonance imaging scan of a patient with ccTGA and dilatation and dysfunction of the systemic right ventricle. **(B)** Main echocardiographic and MRI parameters to study the function of the right ventricle.

In Tetralogy of Fallot, MRI is the gold standard for lifetime imaging (Figure 1A). Echocardiographic parameters such as the tissue Doppler-derived isovolumetric acceleration (IVA) index have been found to correlate with the impaired regional and global longitudinal RV systolic function in a study comparing echocardiographic data with global RV volume and ejection fraction obtained by MRI (18). Comparing echocardiography parameters and MRI data (Figure 1B) remains difficult: parameters obtained by echocardiography such as the myocardial performance index and the isovolumic acceleration index do not correlate with the RV ejection fraction and pulmonary regurgitation fraction derived by cardiac MRI (19).

The RV performance can be reproducibly assessed in patients with Tetralogy of Fallot by measuring the tricuspid annular plane systolic excursion (TAPSE). The correlation of TAPSE with right ventricular ejection fraction (RVEF) is a matter of debate: whereas some groups report a correlation between TAPSE and RVEF in patients with pulmonary arterial hypertension (PAH) associated with CHD (20), other groups recently showed that TAPSE cannot be used to determine RVEF and is not associated with exercise performance in patients after repair for Fallot tetralogy (21).

Echocardiography has been described as reproducible and accurate for assessing RV volumes when compared to MRI measurements. However, additional equipment, such as knowledge based reconstruction models like the VentriPoint System^®^, is needed to obtain these data by echocardiography (22).

Tissue Doppler techniques allow the non-invasive estimation of ventricular filling pressures in patients with single RV. It was found that the strain rate strongly correlates with the invasively measured ventricular end-diastolic pressure (23).

New imaging options using tracer techniques are emerging and will give an opportunity for the simultaneous assessment of cardiac performance at different levels *in vivo*. These techniques will make it possible to assess coronary flow, myocardial perfusion, oxygen delivery, metabolism, and contraction simultaneously (24).

## Surgical Aspects in CHD with RV Pressure/Volume Overload

Surgery in patients with CHD is directed at protecting RV function as long as possible in conditions that present with RV volume and/or pressure overload.

There is evidence suggesting that pulmonary insufficiency and the resulting RV volume overload have detrimental consequences on RV function and clinical parameters such as exercise capacity (25). In addition, RV dilatation due to severe pulmonary regurgitation has been associated with arrhythmias, heart failure, and sudden cardiac death in patients after repair of Fallot tetralogy (26–29).

Although an association of transannular repair in Fallot tetralogy with pulmonary regurgitation and its negative consequences on RV function has been established, the survey published by Al Habib et al. (25) clearly demonstrates that these techniques are still widely used and probably influenced by anatomical conditions (such as a low *z*-score of the pulmonary valve diameter) which limit the surgical techniques available for palliating the situation. In order to answer the question regarding “the best potential repair” in patients with Fallot tetralogy and hypoplastic pulmonary valve and arteries, a large prospective collaborative investigation would be necessary aiming at acquiring follow-up data extending over several decades (25).

Nevertheless, many centers now recommend early pulmonary valve replacement before first symptoms of heart failure will develop (30).

Implantation of a pulmonary valved conduit is now considered a standard procedure for surgical repair of many complex congenital cardiac anomalies with RVOTO and/or pulmonary insufficiency. However, the degeneration of allogeneic and xenogenic pulmonary valve conduits require repeated conduit replacements during a patient’s lifetime. The ideal conduit has not been developed up to now (31).

Xenogenic decellularized tissue-engineered pulmonary valve conduits (TEPVC) recently failed to offer a true advantage and showed a high failure rate (32). Future research is directed at developing autologous valved conduits. Currently, these techniques are under evaluation in animal experiments (33, 34).

The correction of residual anatomic defects (e.g., pulmonary regurgitation in patients with Fallot repair) earlier in life seems appropriate given the pathophysiology of RV deterioration in these patients. However, data are lacking evaluating a more conservative and observant regimen until conduit placement versus a concept with earlier surgical intervention.

Acute RV failure is a major risk factor for mortality of patients after orthotopic heart transplantation when pulmonary vascular disease is present such as in chronic left heart failure. An early mortality rate of 19% has been reported in this subset of patients (35). Reports suggest that the early placement of a RV assist device may serve as a bridge to recovery of patients with RV-failure following heart transplantation (36, 37).

There are new surgical techniques on the horizon aiming at improving RV systolic function in patients with dilated RV after surgery for CHD. Tang and coworkers have recently published a mathematical model analyzing the effects of placing an elastic band in the RV on RVEF (38). Therefore, progress in treatment of RV dysfunction is also expected from modern surgical methods emerging in the future.

## Experimental Research with Focus on RV Dysfunction

Research focusing on RV dysfunction – especially in the context of congenital heart defects – is urgently required. We need to better understand the underlying pathophysiological mechanisms of RV dysfunction in patients with CHD. Even though the RV, when compared to the left ventricle, differs on a morphological, physiological, and molecular level, the RV has gained in scientific interest in the last few years. Extensive work has just begun in order to unravel the etiology of RV dysfunction.

The morphology of the RV, which has a thinner wall than the left ventricle, enables a quick adaptation to changes in preload under physiological conditions. An important mechanism of adaptation of the RV to high pressure is to increase wall thickness by accumulating muscle mass (hypertrophy) and to assume a more rounded shape (39). The cell size is increased due to addition of sarcomeres and an increase in protein synthesis. Protein synthesis in cardiomyocytes is directly induced by stretch and enhanced by autocrine, paracrine, and neurohumoral influences. The increase in afterload is sensed by integrins and stretch-activated ion channels in cardiac cells [myocytes, fibroblasts, endothelial cells (40)]. Integrins are membrane crossing heterodimers that are both firmly attached to the extracellular matrix (ECM) and the cytoskeleton. This allows the transduction of mechanical stress into intracellular chemical signals which are relevant for the synthesis of contractile proteins and proteins for autocrine and paracrine signaling (41, 42). The local response to pressure overload is enhanced in the same way by systemic (neurohumoral) influences, e.g., activation of the renin-angiotensin and sympathetic systems.

The hypertrophic response of cardiomyocytes to pathological conditions leads to changes at the transcriptional level such as an increase of protein synthesis and alterations of fetal genes. Typical markers for fetal gene expression that are merely expressed in fetal ventricles are atrial and brain natriuretic peptides, skeletal α-actin or β-myosin heavy chain (43). It was shown in ovine studies that pressure and volume overload alter the expression levels of myocyte enhancer factor 2, GATA-4, Nkx2.5, transcriptional enhancer factor 1, and specificity protein (Sp) 1 (44). Another elegant study with pulmonary insufficient mice also exhibited altered expression levels of these transcription factors reflecting changes in transforming growth factor (TGF)-β signaling, ECM remodeling, and apoptosis (45). One of the hallmarks of maladaptive cardiac growth is the α- to β-isotype switch of the major thick filament protein myosin heavy chain (MHC) (α-MHC/β-MHC switch) in cardiomyocytes. In pressure overloaded RV associated with PAH, the α-MHC content is reduced from about 30 to ±5% (46). The adenosine triphosphate-ase (ATPase) activity of β-MHC is lower than that of α-MHC. The disappearance of α-MHC results in a significant decrease in systolic function (47). The myocardial regulatory proteins troponin, tropomyosin, and tropomodulin have potential implications in the pathobiology of heart failure (48, 49). Their precise role in failure of the RV in conditions associated with volume overload or increased afterload is yet to be analyzed.

The ECM is an essential part of the matrix scaffold of the heart and consists predominantly of collagen with relatively small amounts of fibronectin, laminin, and elastin. The close proximity of the contractile apparatus implies that the ECM influences the systolic and diastolic function as well as ventricular size and shape (50, 51). Increased collagen content of the heart (fibrosis) is tightly linked to TGF-β1. In addition, the excessive degradation of the matrix by matrix metalloproteinases (MMPs) will adversely affect myocardial systolic and diastolic function (50, 52, 53). The TGF-β1 and MMP signaling are linked in a complex fashion (52). Experimental (53) and clinical (46) data show increased fibrosis in the myocardium under conditions of increased pulmonary afterload. At present it remains unclear to what extent these changes are related to RV ischemia and disturbed RV microcirculation. Most of the studies investigating these problems have been conducted with left ventricular strain and failure models, but it is not known how extensively these mechanisms contribute to RV failure.

Angiotensin II (Ang II), Endothelin-1 (ET-1), and other neurohormones are able to induce hypertrophic pathways within the cardiomyocyte, in part, by formation of reactive oxygen species (ROS) (54). In left ventricular failure, increased generation of ROS and reactive nitrogen species (RNS) and a reduced activity of cytoprotective enzymes are documented (55). The contribution of ROS and RNS to RV failure has to be investigated.

Neutrophils may be an important source of excessive ROS formation in heart failure. In parallel with the recruitment of immune cells, proinflammatory cytokines play a complex role in the development of heart failure. Hemodynamic and clinical parameters of disease severity correlate with increased serum levels and expression of the proinflammatory cytokines tumor necrosis factor (TNF)-α, interleukin (IL)-1, and IL-6 by cardiomyocytes (56). Recent data demonstrate that TNF-α has a pivotal role in adverse myocardial remodeling and inhibition of TNF-α. The binding protein etanercept can attenuate the progression to heart failure in experimental volume overload (57). A recently detected IL-1-related protein, IL-33, is a functional ligand of the IL-1 receptor family member [IL-1 receptor – like protein transmembrane isoform (ST2L)] which is produced by cardiac fibroblasts in response to mechanical strain (58). Increased levels of IL-1 receptor-like protein soluble isoform (sST2) predict mortality and transplantation in patients with chronic heart failure, independent of B-type natriuretic peptide (BNP) or atrial natriuretic peptide (proANP) levels (59). Hitherto, there is no information on the precise role of cardiac inflammation and immune activation in the failing RV. Recent evidence suggests that stimulation of NADPH oxidases (a known target of Ang II and ET-1) and ROS formation may be of critical importance in mediating the slow cardiac response to stretch. The positive inotropic effect of ET-1 requires intact NADPH oxidase activity and protein kinase A (PKA) signaling. The latter may be directly activated by ROS formation, opening the possibility that the NO-redox state of the myocardium may directly affect protein phosphorylation and the inotropic state, independent of adrenergic receptor stimulation (60).

In conditions with increased afterload (i.e., in pulmonary hypertension) the systolic right coronary artery flow is reduced as assessed by MRI (61). There is little information how the RV branches of the right coronary artery adapt to RV remodeling and chronically increased wall stress. Current suggestions favor a similar pattern as observed in the left ventricle: increased oxygen extraction at rest and a higher dependence on increased coronary flow to meet increased myocardial oxygen demand. During cardiac hypertrophy, a mismatch between the numbers of capillaries and the size of the cardiomyocytes can result in myocardial hypoxia, contractile dysfunction, and apoptosis (62). It is not known at present whether microvessels in RV hypertrophy vanish or whether angiogenesis matches the degree of hypertrophy. RV capillary density and vascular endothelial growth factor (VEGF) expression are increased in chronic hypoxic pulmonary hypertension (63).

While cardiomyocyte apoptosis is rare in the normal heart (1 apoptotic cardiomyocyte in 10^4^ to 10^5^ cells) (64), it has been shown that the apoptosis rate increases up to 1 in 400 cardiomyocytes in human heart failure (65). In the rat model of pulmonary artery banding, the apoptosis rate of RV cardiomyocytes has been shown to be elevated (66). In the mouse model apoptosis has been related to lethal dilated cardiomyopathy (67). There is no information on the impact of chronic RV volume overload on RV cardiomyocyte apoptosis available at present. Blocking apoptosis using broad spectrum caspase inhibitors has been shown to reduce LV ischemia reperfusion injury in the experimental setup (68, 69), but currently there is no information on the effect of such treatment on RV structure and function.

Notable analyses of gene expression patterns and epigenetic modifications during maladaptive processes in the heart revealed differences between the left and RV (70, 71). Thus, the cardiac biomarkers BNP and ANP have distinct expression patterns in the left and RV of mouse hearts after epigenetically modifications on their promoter regions, such as acetylations and methylations (72). Furthermore, studies with rabbits could impressively demonstrate transcriptional and translational differences between the two chambers of the heart after banding of the thoracic aorta and the pulmonary artery, respectively. Manipulations resulted in altered expression profiles of genes which are involved in energy metabolism, signaling pathways associated with actin, Integrin-linked kinase (ILK), calcium handling, or cardiac tissue development (73). MicroRNAs (miRNAs) get more and more into the center of attention in cardiovascular research. Several studies provided information about dysregulated miRNAs, such as miRNA-195, miRNA-208, and miRNA-133 in hypertrophic hearts. This evidence may offer new therapeutic opportunities (74).

**Figure 2:**
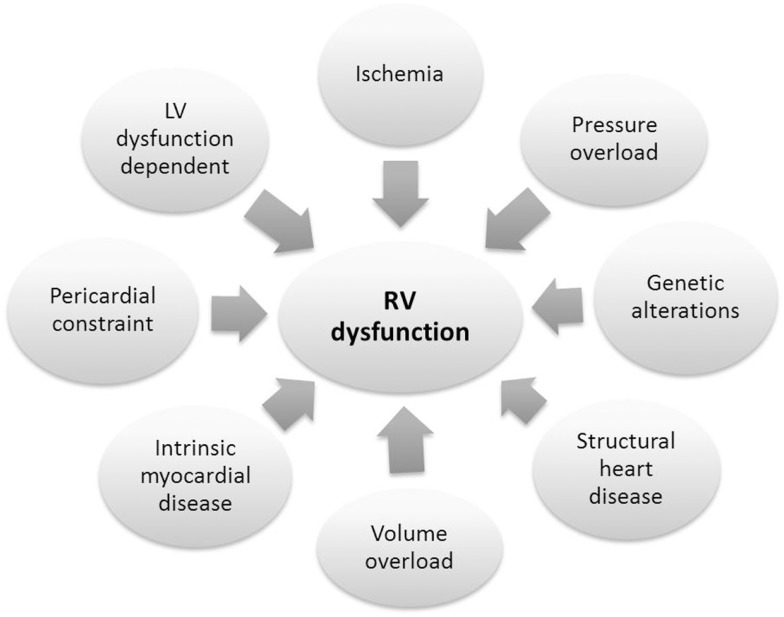
**Main mechanisms of right ventricular dysfunction**.

Congenital heart defects and other diseases associated with RV impairment show a wide variety of causes (Figure 2). Many genes and complex signaling pathways are involved in heart development and processes of heart adaption during altered conditions. So far, efforts in research on RV pathomechanisms had been focused on PAH (75) underscoring the lack of studies concerning other diseases of the right heart. There is an incredible lack in exploring disease mechanisms caused by volume overload on the RV which is especially of importance after the correction of Tetralogy of Fallot.

Until now, there is merely one study known about volume overload of the RV in a small animal model, possibly due to technical difficulties in implementation. Reddy and colleagues generated the first murine model for pulmonary insufficiency and analyzed its physiologic and molecular characteristics. In their model, RV volume overload resulted in declined heart function and altered expression of various genes that are involved in mitochondrial and G protein-coupled receptor signaling (45). In contrast, few studies with large animal models exist. Recently, researchers analyzed the effects of volume overload (by pulmonary artery shunting) and pressure overload (by pulmonary artery banding), respectively, on the RV in fetal lambs. All animals showed altered expression levels of the transcriptional activators MEF-2, GATA-4, Nkx2.5, SP-1, and SP-3, which are important for early developmental processes (44). In contrast to shunting in lambs, RV volume overload could also be produced by pulmonary insufficiency, e.g., by surgical techniques such as a suture plication of the pulmonary valve leaflets in piglets (76) or in sheep by the widening of the RV outflow tract using a transannular patch (77). In conclusion, scientists should develop models to better understand the pathological processes in the RV as a lot of questions still remain unanswered.

Besides the great advantage of large animal models because of their better comparability to human disease settings and easier implementation of operation techniques, large animals produce high costs and are more laborious in keeping. One of the most important strategies to uncover the etiology of diseases associated with the right heart will be the use of genetic approaches and small animal models. Even conspicuous is the predominant utilization of rodent animal models such as mice and rats based on their many advantages like the presence of a four chambered heart and an extensive knowledge of their genetics (75). The most commonly used rat models to study pulmonary hypertension apply techniques such as monocrotaline (78), hypoxia (79), constriction of the pulmonary artery by banding or clipping (80, 81). Fawn hooded rats spontaneously develop a PH phenotype (82).

Current efforts in studying RV pathomechanisms in the setting of experimental PAH generated new interesting findings. Sutendra and colleagues could show that in monocrotaline treated rats the earlier described metabolic shift is not sustained during the development of RV failure (78). Furthermore, Carvedilol is a substance known to reduce RV hypertrophy in experimental PAH. Drake and colleagues analyzed the effect of Carvedilol treatment on rats after development of RV failure using chronic hypoxia and treatment with a VEGF receptor inhibitor. This study displayed an altered gene expression profile thereby revealing the mode of action and mechanisms of RV failure (79).

All these models, however, could not reflect the real clinical presentation of patients with RV dysfunction. The strengths and weaknesses of various animal models emphasize the urgent need for better models to uncover the disease mechanisms in the RV. Mice overexpressing signaling components such as serotonin transporter (SERT), bone morphogenetic protein receptor-2 (BMPR-2), or S100A4 that are involved in right heart failure could only demonstrate details of maladaptive processes in the right heart. More detailed analyses such as investigations in the RV specific ubiquitin-proteasome signaling (83) or analyses of the role of components like cyclooxygenase 2 (84) are needed for gaining deeper insights into right heart diseases. Besides metabolic shifting, ECM remodeling processes are of further interest in research of RV failure in congenital heart defects. For instance, the maladaptive increase of ECM components in the RV of patients in the first weeks after heart transplantation can be found reduced after 3 years (85) underlining the importance of this topic in cardiology. Walker and colleagues discovered changes in the phosphorylation of key contractile proteins, like troponin T, myosin-binding protein C (MBP-C), and myosin light chain 2 (mlc2) under the setting of pressure overloading in calves (86). These results show once again that RV pathological mechanisms differ from those of the left ventricle, but the knowledge of RV disease mechanisms is still insufficient. A lot of open questions remain such as why metabolic remodeling occurs or whether the immune response in PAH patients is the cause or consequence of the disease (87).

## Conclusion

In the future, RV dysfunction will represent a growing problem due to the increasing number of patients with CHD surviving into adulthood with the morphological RV serving as the systemic ventricle. In addition, patients after pulmonary artery conduit implantation are at risk for developing RV dysfunction due to volume or/and pressure overload during their whole lifetime.

At present, most knowledge of RV dysfunction is mainly derived from diseases of the left ventricle. Experimental work has to be performed to obtain more insight into the pathophysiology of RV dysfunction. Modern imaging techniques such as molecular imaging may give more information on the myocardial metabolism during RV dysfunction. Current pharmacological treatment is based on a small panel of drugs, which exert no right heart specific properties. Therefore, future research has to be focused on developing a targeted drug therapy for these patients.

We expect major improvements in the therapy of RV dysfunction by technologies that will enable the placement of smaller ventricular assist devices in patients presenting with acute and chronic right heart failure. Stem cell therapy is another promising therapeutic approach worth mentioning.

However, the knowledge gained by translational and molecular biological research will lead to a more targeted therapy for treating the failing RV.

## Authors Contribution

Matthias Gorenflo was responsible for the concept of this manuscript and contributed to major parts of the manuscript. Raoul Arnold contributed to the imaging section. Tsvetomir Loukanov contributed to the section on surgical therapy. Doreen Köhler was responsible for the experimental section of this manuscript and for editing the manuscript.

## Conflict of Interest Statement

The authors declare that the research was conducted in the absence of any commercial or financial relationships that could be construed as a potential conflict of interest.
